# From *Cannabis sativa* to Cannabidiol: Promising Therapeutic Candidate for the Treatment of Neurodegenerative Diseases

**DOI:** 10.3389/fphar.2020.00124

**Published:** 2020-03-06

**Authors:** Tommaso Cassano, Rosanna Villani, Lorenzo Pace, Antonio Carbone, Vidyasagar Naik Bukke, Stanislaw Orkisz, Carlo Avolio, Gaetano Serviddio

**Affiliations:** ^1^Department of Clinical and Experimental Medicine, University of Foggia, Foggia, Italy; ^2^Department of Medical and Surgical Sciences, University of Foggia, Foggia, Italy; ^3^Department of Physiology and Pharmacology “V. Erspamer,” Sapienza University of Rome, Rome, Italy; ^4^Morphological Science Department of Human Anatomy, Medical Faculty University of Rzeszów, Rzeszów, Poland

**Keywords:** *Cannabis sativa*, oxidative stress, phytocannabinoids, cannabidiol, Alzheimer's disease, Parkinson's disease

## Abstract

*Cannabis sativa*, commonly known as marijuana, contains a pool of secondary plant metabolites with therapeutic effects. Besides Δ9-tetrahydrocannabinol that is the principal psychoactive constituent of *Cannabis*, cannabidiol (CBD) is the most abundant nonpsychoactive phytocannabinoid and may represent a prototype for anti-inflammatory drug development for human pathologies where both the inflammation and oxidative stress (OS) play an important role to their etiology and progression. To this regard, Alzheimer's disease (AD), Parkinson's disease (PD), the most common neurodegenerative disorders, are characterized by extensive oxidative damage to different biological substrates that can cause cell death by different pathways. Most cases of neurodegenerative diseases have a complex etiology with a variety of factors contributing to the progression of the neurodegenerative processes; therefore, promising treatment strategies should simultaneously target multiple substrates in order to stop and/or slow down the neurodegeneration. In this context, CBD, which interacts with the eCB system, but has also cannabinoid receptor-independent mechanism, might be a good candidate as a prototype for anti-oxidant drug development for the major neurodegenerative disorders, such as PD and AD. This review summarizes the multiple molecular pathways that underlie the positive effects of CBD, which may have a considerable impact on the progression of the major neurodegenerative disorders.

## Introduction

Oxidative stress (OS) plays a crucial role in aging and occurs manly when the activity of the anti-oxidants enzymes is not sufficient to counterbalance the generation of reactive oxygen species (ROS). In the latter condition, high production of ROS can alter the structure of proteins, lipids, nucleic acids, and matrix components leading to programmed cell death (Cassano et al., 2016). Different tissues present different susceptibility to OS. The central nervous system (CNS) is extremely sensitive to this type of damage for several reasons. To this regard, the CNS has a low level of antioxidant enzymes, a high content of oxidizable substrates, and a large amount of ROS produced during neurochemical reactions (Trabace et al., 2004; Uttara et al., 2009). In addition to several other environmental or genetic factors, OS contributes to neurodegeneration since free radicals attack neural cells. Therefore, neurons suffer a functional or sensory loss during the neurodegenerative process. Even if oxygen is indispensable for life, an unbalanced metabolism and an excess production of ROS ends up in a series of pathological conditions, such as Alzheimer's disease (AD), Parkinson's disease (PD), and many other neural disorders. Free radicals cause lesions to protein and DNA, activate inflammatory process and subsequent cell apoptosis (Cassano et al., 2012).

In the last years, there is an urgent need to discover new drug targets that can effectively combat cell alteration caused by the stress of cell membranes. In this perspective, the endocannabinoid (eCB) system has attracted considerable interest due to the current interplay between eCB and different redox-dependent signaling pathways. The two well-characterized eCBs are N-arachidonoyl-ethanolamine or anandamide (AEA) and 2-arachidonoyl-glycerol (2-AG), which are synthesized on demand in response to elevations of intracellular calcium (Howlett et al., 2002; Di Marzo et al., 2005) and respectively metabolized by fatty acid amide hydrolase (FAAH) and monoglyceride lipase (MAGL) (Piomelli, 2003; Di Marzo, 2008; Kunos et al., 2009). Cannabinoid (CB) receptors exist in two different subtypes: type 1 (CB1) and type 2 (CB2) (Matsuda et al., 1990; Munro et al., 1993; Howlett et al., 2002). The CB1 receptors, first cloned in 1990, are widely distributed in the body and in the CNS are distributed at the level of basal ganglia, cerebellum, hippocampus, caudate nucleus, putamen, hypothalamus, amygdala, and spinal cord (Matsuda et al., 1990). The CB2 receptors, cloned in 1993, are mainly located in cells of the immune system with high density in the spleen, T lymphocytes, and macrophages (Munro et al., 1993). Their anatomical distribution correlates them to the actions for which they are responsible: the activation of the CB1 receptors has euphoric effects and an antioxidant, antiemetic, analgesic, antispasmodic, and appetite stimulating actions. As for CB2 receptors, their stimulation is attributable to the anti-inflammatory and immunomodulatory actions of CB (Cassano et al., 2017).

Converging evidence strongly suggests that eCBs act as retrograde synaptic messengers (Kano et al., 2002; Freund et al., 2003). This phenomenon is initiated postsynaptically by an elevation of cytoplasmic calcium concentration that induces the production and release into the synaptic space of eCBs. Thereafter, eCBs activate CB1 receptors at presynaptic levels and block the release from the terminals of neurons of different transmitters, such as gamma-aminobutyric acid (GABA), glutamate, dopamine (DA), noradrenaline, serotonin, and acetylcholine (Howlett et al., 2002; Pertwee and Ross, 2002; Szabo and Schlicker, 2005). These mechanisms mediated by the activation of presynaptic CB1 receptors are termed depolarization-induced suppression of inhibition (DSI) and excitation (DSE), respectively when are involved the inhibitory (GABA) or excitatory (glutamate) synaptic transmissions (Kano et al., 2002; Freund et al., 2003). Likewise, CB2 receptors can modulate the production and function of certain inflammatory cytokines at multiple levels by activating the immune cells and modulating their migration both within and outside the CNS (Freund et al., 2003; Walter and Stella, 2004). Antioxidant enzymes can be modulated by eCBs, not only acting on the CB1 and CB2 receptors, but also through the transient receptor potential vanilloid-1 (TRPV1), the peroxisome proliferator-activated receptor alpha (PPAR-alpha), and the orphan receptors N-arachidonyl glycine receptor or G-protein-coupled receptors 18 (GPR18) GPR19 and GPR55 (Piomelli, 2003; McHugh et al., 2010; Howlett et al., 2011; McHugh, 2012). Therefore, the direct and/or indirect modulation of pathways through which the eCBs damper the OS may represent a promising strategy for reducing the damage caused by a redox imbalance (Gallelli et al., 2018). Moreover, antioxidants are now seen as a convincing therapy against severe neurodegeneration, as they have the ability to fight it by blocking the OS. Diet and medicinal herbs are an important source of antioxidants. The recognition of antioxidant therapy upstream and downstream of OS has proven to be an effective tool to improve any neuronal damage as well as to eliminate free radicals. Antioxidants have a wide field of application and can prevent OS interacting with the metal ions, which play an important role in the build-up of neuronal plaque (Uttara et al., 2009).

In the last decade there are increasing evidences that secondary plant metabolites, extracted from medicinal herbs, may represent lead compounds for the production of medications against inflammation and OS, protecting from neuronal cell loss (Giudetti et al., 2018). Among these medicinal herbs, *Cannabis sativa*, commonly known as marijuana, contains a pool of secondary plant metabolites with therapeutic effects (Gugliandolo et al., 2018). In this context, cannabidiol (CBD) the nonpsychotropic CB extract from *Cannabis sativa* may represent a prototype for anti-inflammatory drug development for those human pathologies where both the inflammation and OS play a key role to their etiology and progression (Izzo et al., 2009). To this regard, therapies that effectively combat disease progression are still lacking in the field of neurodegenerative disorders, and mostly with AD. CBD, which modulates the eCB system, but has also CB receptor-independent mechanism, seems to be a prototype for anti-inflammatory drug development.

Therefore, the present review summarizes the main molecular mechanisms through which CBD exerts its beneficial effects that may have a considerable impact on the progression of the major neurodegenerative disorders.

### Cannabis sativa

The medical and psychotropic effects of *Cannabis sativa* have been well known since long time. A multitude of secondary metabolites was extracted from this plant and most of them were used for therapeutic purpose by many cultures. So far more than 400 chemical compounds have been isolated from *Cannabis sativa* and among them more than 100 terpeno-phenol compounds named phytocannabinoids have been detected (Mechoulam and Hanus, 2000; Mechoulam et al., 2007). As such *Cannabis sativa* can be regarded as a natural library of unique compounds. The most abundant phytocannabinoid is the Δ9-tetrahydrocannabinol (delta-9-THC), responsible for the psychotropic effect associated with *Cannabis* consumption, and then the nonpsychoactive constituent CBD and cannabigerol (CBG) (Mechoulam and Hanus, 2000; Mechoulam et al., 2007; Gugliandolo et al., 2018). **Table 1** shows the list of the most abundant nonpsychoactive phytocannabinoids isolated from *Cannabis sativa*. Phytocannabinoids mimic the effects of eCBs that regulate the transmission of nerve impulses in some synapses of the nerve pathways, causing in particular a reduction in the release of signals between the cells (Piomelli, 2003).

**Table 1:** Most abundant nonpsychoactive phytocannabinoids isolated from *Cannabis sativa*: chemical structures and pharmacological actions.

Phytocannabinoids	Mechanisms	Effects	References
	CB_2_ inverse agonist	Anti-inflammatory effects	Thomas et al., 2005
	CB_1_, CB_2_ antagonist	Antispasmodic effect	
	FAAH inhibition	Reduces FAAH expression in the inflamed intestine	Ligresti et al., 2006
	TRPA1 agonist	Analgesic effects	De Petrocellis et al., 2008
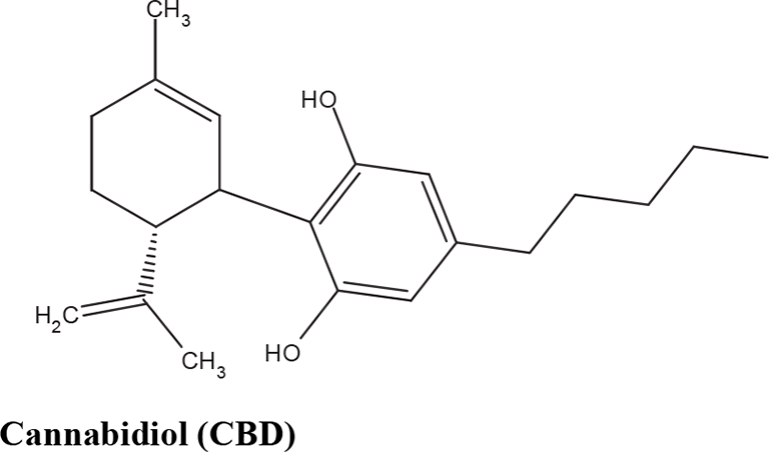	TRPM8 antagonist	Analgesic effects. Potential role in prostate carcinoma	
	TRPV1 agonist	Antipsychotic and analgesic effects	
	Adenosine uptake competitive inhibitor	Anti-inflammatory effects	Carrier et al., 2006
	PPARγ agonist	Vasorelaxation and stimulation of fibroblasts into adipocytes	O'Sullivan et al., 2009
	5HT_1A_ agonist	Anti-ischemic and anxiolytic properties	Campos and Guimarães, 2008 Resstel et al., 2009
	Ca^2+^ channel	Neuroprotective and antiepileptic properties	Drysdale et al., 2006 Ryan et al., 2009
	Suppressor of tryptophan degradation	Potential role in pain, inflammation and depression	Jenny et al., 2009
		
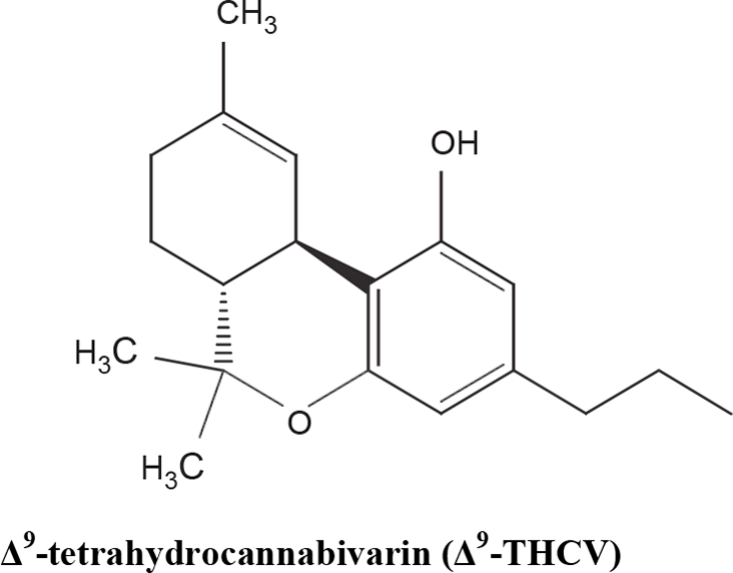	CB_1_ antagonist	Increases central inhibitory neurotransmission	Thomas et al., 2005 Dennis et al., 2008 Ma et al., 2008
	CB_2_ partial agonist	Stimulates mesenchymal stem cells	Scutt and Williamson, 2007
		
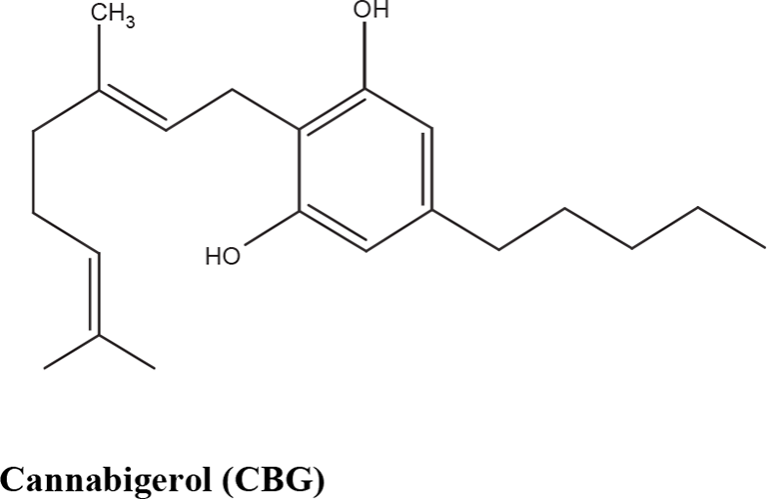
	TRPV1 agonist	Potential role in analgesia	Ligresti et al., 2006
	TRPA1 agonist		De Petrocellis et al., 2008
	TRPM8 antagonist		
		
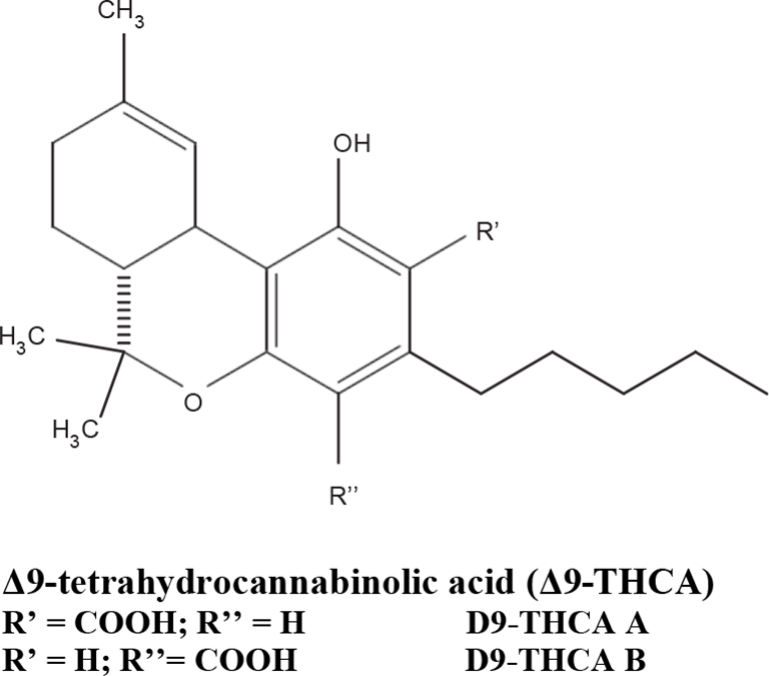	TRPA1 partial agonist	Potential role in analgesia	De Petrocellis et al., 2008
	TRPM8 antagonist		
		
		
		
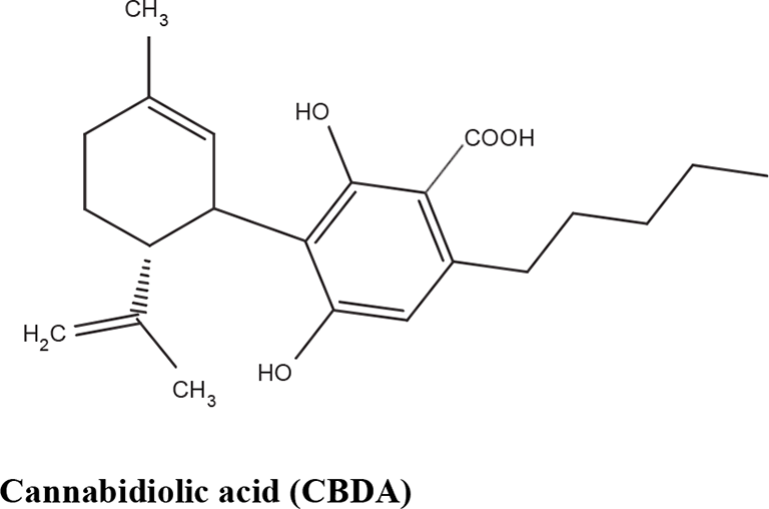	TRPA1 partial agonist		De Petrocellis et al., 2008
	TRPM8 antagonist	Potential role in analgesia	
	TRPV1 agonist		Ligresti, A., et al., 2006
	COX-2 inhibitor	Potential role in inflammation	Takeda et al., 2008

Due to its high lipophilicity and its affinity for lipid membranes, delta-9-THC was supposed to bind non-specifically variety of cell membranes modifying their fluidity rather than to activate a specific receptor (Hillard et al., 1985). Later this first hypothesis was completely discarded and was demonstrated that delta-9-THC exerts its effects by combining with a selective receptor (Devane et al., 1988; Howlett et al., 1990; Matsuda et al., 1990). In fact, many authors have demonstrated that delta-9-THC exerts its psychoactive effects acting on CB1 receptors, whereas CDB and CBG, two nonpsychoactive CBs, have low affinity for both CB1 and CB2 receptors and inhibit FAAH, resulting in increased levels of eCBs, which in turn further activate the CB1 receptor (Devane et al., 1988; Howlett et al., 1990; Matsuda et al., 1990; Appendino et al., 2011). Among the nonpsychoactive phytocannabinoids, most of the evidences have focused on CBD, which possesses a high antioxidant and anti-inflammatory activity, together with neuroprotective, anxiolytic and anticonvulsant properties (Pellati et al., 2018).

### Mechanisms of CBD Action

After delta-9-THC, CBD is the second most abundant phytocannabinoids and is one of the major nonpsychoactive CB constituents in the plant of *Cannabis sativa* representing up to 40% of *Cannabis* extract. Adams and colleagues first isolated the CBD, while Mechoulam and colleagues analyzed its structure and stereochemistry (reviewed in Pertwee, 2006). Therapeutically CBD is already available alone and in formulation with delta-9-THC (Booz, 2011). In particular, a drug containing only CBD (Epidiolex) is used for children affected by epilepsy resistant to other treatments, as well as in combination (1:1 ratio) with delta-9-THC (CBD/delta-9-THC, Sativex/Nabiximols) is currently used to treat the spasticity observed in patients affected by multiple sclerosis (Pertwee, 2008; Devinsky et al., 2016). Compared to delta-9-THC, CBD possess a better safety profile and it is well tolerated when administered at animals and patients even at high doses (up to 1,500 mg/day) (Bergamaschi et al., 2011). In fact, authors demonstrated that CBD did not alter cardiovascular parameters, body temperature, psychomotor, and psychological functions, as well as did not induce catalepsy like delta-9-THC (Bergamaschi et al., 2011). Unlike delta-9-THC, CBD does not target directly the CB receptors and this characteristic may justify its better safety profile compared to delta-9-THC (Pertwee, 2006; Thomas et al., 2007).

Although the pharmacodynamics of CBD is not fully clarified, different evidences have been accumulated showing that CBD seems to act throughout different pathways. To this regard, although CBD shows much lower affinity than delta-9-THC for CB1 and CB2 receptors, it is able to antagonize CB1/CB2 receptor agonists *in vitro* at reasonably low concentrations (nanomolar range) (Thomas et al., 2007). In particular, it has been shown by *in vitro* studies that CBD is able to act as CB1/CB2 receptors inverse agonist an action that underlies its antagonism of CP55940 and R-(+)-WIN55212 at the CB1/CB2 receptor (Thomas et al., 2007). It has been hypothesized that the anti-inflammatory actions of CBD might be due to its ability to act as a CB2 receptor inverse agonist (Pertwee, 2006). Besides CB receptors, CBD has been profiled also towards other pharmacological substrates. To this regard, CBD showed also affinity to the peroxisome proliferation-activated receptors (PPARs), which are a family of ligand-inducible transcription factors that belong to the nuclear hormone receptor superfamily. In humans, there are three PPAR isoforms PPARα, PPARβ/δ, and PPARγ that are encoded by separate genes and are differently expressed in organs and tissues (Michalik et al., 2006). CBD seems to activate the transcriptional activity of PPARγ, which play a primary role in the regulation of adipocyte formation, insulin sensitivity and activation of inflammatory response (O'Sullivan, 2007; O'Sullivan and Kendall, 2010; Hind et al., 2016; O'Sullivan, 2016). To this regard, CBD activates PPARγ receptors leading to a lower expression of proinflammatory genes, which were inhibited by PPARγ antagonists (Esposito et al., 2007; Esposito et al., 2011; O'Sullivan, 2016).

Moreover, CBD exerts a more potent antioxidant effects than other antioxidants, such as ascorbate or α-tocopherol, in *in vitro* study where cortical neurons were treated with toxic concentrations of glutamate (Hampson et al., 1998; Campos et al., 2016).

The neuroprotective effect was present regardless of whether the insult was due to the activation of N-methyl-D-aspartate (NMDA) receptor, α-amino-3-hydroxy-5-methyl-4-isoxazolepropionic acid (AMPA) receptor, or kainate receptors and, more interestingly, it was not mediated by CB receptors since the CB antagonist was unaffected (Hampson et al., 1998). The latter result suggests that CBD may be a potent antioxidant without psychotropic side effects, which are mediated by the direct action on CB receptors.

The anti-inflammatory effect of CBD is also mediated by the adenosine A_2A_ (A_2A_) receptor whose activation dampers the immune system, leading to a reduction of the antigen presentation, immune cell trafficking, immune cell proliferation, production of the proinflammatory cytokine, and cytotoxicity (Magen et al., 2009). In fact, it has been shown that CBD enhances A_2A_ receptor signaling by the inhibition of cellular update of an adenosine transporter leading to anti-inflammatory and antioxidant effects (Carrier et al., 2006). Likewise, CGS-21680, which is an agonist of the A_2A_ receptor, mimics the actions of CBD that were suppressed by an A_2A_ antagonist (i.e. ZM241,385) (Martín-Moreno et al., 2011).

The CBD neuroprotective property seems to be due also to the activation of 5-hydroxytryptamine subtypes 1A (5-HT1A) receptors, which are located in pre- and post-synaptic membranes in several brain regions (Hoyer et al., 1986). Russo and colleagues first demonstrated that CBD is able to activate the 5-HT1A receptors (Russo et al., 2005). Further support to this first observation was given by a recent study where the authors found that the effect of CBD was blocked by WAY-100135, a selective 5-TH1A receptor antagonist (Galaj et al., 2019).

Finally, it has been demonstrated that CBD has a direct effect on mitochondria (da Silva et al., 2018). To this regard, it has been widely accepted that mitochondrial dysfunction can contribute to neurodegeneration due to the overproduction of ROS and iron accumulation (Mills et al., 2010; Serviddio et al., 2011; Cassano et al., 2012; Cassano et al., 2016; Romano et al., 2017). In particular, iron overload induces several mitochondrial alterations, such as increased mitochondrial DNA (mtDNA) deletions and reduction of epigenetic mtDNA modulation, mitochondrial ferritin levels, and succinate dehydrogenase activity, which may altogether alter cellular viability leading to neurodegenerative process (da Silva et al., 2018). Interestingly, all these iron-induced mitochondrial alterations were completely reversed by CBD, which promotes neural cell survival (da Silva et al., 2018). Moreover, doxorubicin, a broad-spectrum chemotherapeutic drug, induces a dose-dependent cardiotoxicity through the dysregulation of various metabolic signaling pathways, including mitochondrial dysfunction (Hao et al., 2015). In particular, doxorubicin reduces the activity of myocardial mitochondrial complexes (I and II) and glutathione peroxidase leading to an increase of ROS generation (Hao et al., 2015). Interestingly, CBD significantly attenuated doxorubicin-induced cardiotoxicity and cardiac dysfunction by improving mitochondrial complex I activity and enhancing mitochondrial biogenesis (Hao et al., 2015).

**Figure 1:**
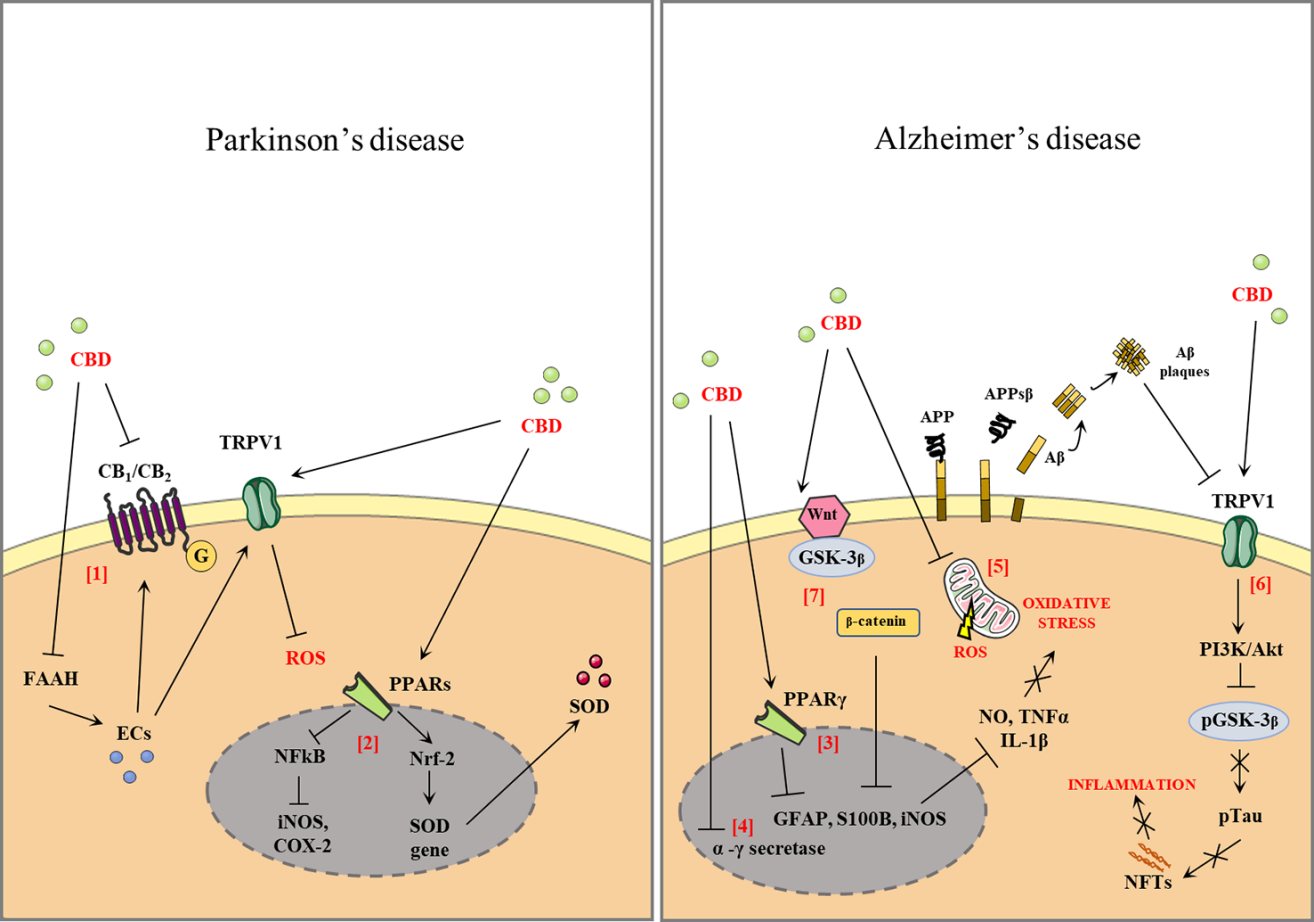
Effect of cannabidiol (CBD) in Parkinson's disease and Alzheimer' disease (AD). CBD antagonizes the action of cannabinoid receptors (CB1, CB2) acting as a reverse agonist and negative allosteric modulator of both receptors. CBD also inhibits fatty acid amide hydrolase (FAAH), resulting in increased levels of endocannabinoids (ECs). ECs activate the anti-oxidant and anti-inflammatory effects that are partially mediated by the actions of the CBD of transient receptor potential cation channel subfamily V member 1 (TRPV1) [1]. CBD binds the peroxisome proliferator-activated receptors (PPARs), antagonizes the action of nuclear factor kappa-light-chain-enhancer of activated B cells (NFkB), and reduces the expression of proinflammatory enzymes such as inducible nitric oxide synthases (iNOS), cyclooxygenase-2 (COX-2), and proinflammatory cytokines [2]. Activation of PPARγ by modulating the expression of proinflammatory mediators such as nitric oxide (NO), tumor necrosis factor α (TNF-α), interleukin 1β (IL-1β), interleukin 6 (IL-6), iNOS, and COX-2 [3]. The CBD downregulates the β- and γ-secretase genes leading to a reduction in amyloid-β (Aβ) production [4]. CBD is able to reduce the oxidative stress (OS) through the attenuation of mitochondrial dysregulation and reactive oxygen species (ROS) generation or by the decrease of the expression of several ROS generating nicotinamide adenine dinucleotide phosphate (NADPH) oxidase isoforms [5]. The stimulation of transient receptor potential vanilloid-1 (TRPV1) by CBD can activate phosphoinositide 3-kinases/protein kinase B (PI3K/Akt) signaling, which in turn inhibits glycogen synthase kinase 3 β (GSK-3β) by phosphorylation of Ser9, thus reducing tau phosphorylation [6]. CBD reduces the activity of p-GSK-3β, the active phosphorylated form of GSK3-β, and causes an increase in the Wnt/β-catenin pathway. The activation of this pathway can protect against OS and Aβ neurotoxicity in AD [7].

Since CBD targets multiple substrates, it may be a good candidate as a multimodal drug for the major neurodegenerative disorders, such as PD and AD. **Figure 1** shows the effects of CBD in PD and AD.

### CBD and PD

PD is a progressive neurodegenerative disorder characterized manly by motor alterations, such as akinesia, bradykinesia, tremors, postural instability, and rigidity. Although the etiology of PD is still largely elusive, its pathophysiology is characterized by loss of midbrain substantia nigra DA neurons and overwhelming evidence indicates that OS is a central factor in PD pathophysiology (Hirsch et al., 1988; Branchi et al., 2010; Aureli et al., 2014). It has been demonstrated in animal model of PD that CBD exerts a neuroprotective effect as antioxidant compound acting through a mechanism that is CB receptor-independent (Fernández-Ruiz et al., 2013). In fact, in 6-hydroxydopamine-lesioned mice CBD was able to significantly reduce the DA depletion and to attenuate the OS increasing the expression of Cu,Zn-superoxide dismutase (SOD), which is an important endogenous mechanism that defences cell against OS (Fernández-Ruiz et al., 2013; Martinez et al., 2015). The latter evidence indicates that CBs having antioxidant CB receptor-independent properties attenuate the neurodegeneration of nigrostriatal dopaminergic fibers occurring in PD (García-Arencibia et al., 2007). This thesis is reinforced by the observation that CBD reduces the neuronal cell death in the striatum occurring after the administration of 3-nitropropionic acid (3NP), an inhibitor of mitochondrial complex II. In particular, the authors demonstrated that 3NP administration causes a reduction of both GABA levels and striatal atrophy of the GABAergic neurons as indicated by a depletion of mRNA levels of proenkephalin (PENK), substance P (SP), and neuronal-specific enolase (NSE). Moreover, the inhibition of mitochondrial complex II induced by 3NP reduces the mRNA expression of superoxide dismutase-1 (SOD-1) and -2 (SOD-2), which are endogenous defences against the OS. Interestingly, after 3NP administration CBD can completely abolish the atrophy of the GABAergic neurons and significantly increase the mRNA levels of SOD-2, as well as attenuate the reduction of mRNA levels of SOD-1 and PENK. Differently, after 3NP administration the administration of arachidonyl-2-chloroethylamide (ACEA) or HU-308, respectively agonist of CB1 and CB2 receptor, did not revert the striatal atrophy of the GABAergic neurons, as well as did not restore the endogenous defences against the OS induced by 3NP (Sagredo et al., 2007). Taken together, these results suggest that CBD exerts a neuroprotective role on the GABAergic neurons that project from the striatum to the substantia nigra and further confirm that its mechanism is CB receptor-independent (Sagredo et al., 2007). Furthermore, in another study authors explored whether CBD was able to attenuate the pathological symptoms of PD modulating the GPR55. In particular, mice were treated for 5 weeks with 1-methyl-4-phenyl-1,2,3,6-tetrahydropyridine/probenecid (MPTPp), which induced motor function impairment and loss of tyrosine hydroxylase-positive neurons and DA levels in the brain. This chronic mouse model of PD was treated with abnormal-CBD (Abn-CBD), a synthetic CBD isomer and GPR55 agonist. Authors found that the key features of PD induced by MPTPp were prevented by the pharmacological treatment, suggesting that the activation of GPR55 may be a good strategy for the treatment of PD (Celorrio et al., 2017).

### CBD and AD

AD is the most common form of dementia affecting elderly people and its pathology is characterized by the accumulation of amyloid-β (Aβ) plaques and tau neurofibrillary tangles (NFTs) in the brain (Querfurth and LaFerla, 2010).

Although the etiology of AD appears to be linked to a multitude of mechanisms, inflammation seems to play a crucial role in its pathogenesis (Bronzuoli et al., 2018; Scuderi et al., 2018). Expected benefits of current therapies are limited (Sabbagh, 2009; Neugroschl and Sano, 2010), so that there is pressing demand for discovering new treatments able to slow disease progression or prevent its onset.

In this contest, the anti-inflammatory properties of CBD were evaluated by both *in vitro* and *in vivo* studies in an animal model of Aβ-induced neuroinflammation (Iuvone et al., 2004; Esposito et al., 2006; Esposito et al., 2007; Esposito et al., 2011). In particular, authors demonstrated that CBD reduces the tau protein hyperphosphorylation through the inhibition of Wingless-type MMTV integration site family member (Wnt) pathways and significantly attenuates all the markers of the Aβ-induced neuroinflammation, including the glial fibrillary acidic protein (GFAP) and inducible nitric oxide synthase (iNOS) protein expression, nitrite production, and interleukin 1 β (IL-1β) (Iuvone et al., 2004; Esposito et al., 2006; Esposito et al., 2007; Iuvone et al., 2009; Esposito et al., 2011). CBD pre-treatment induces a reduction of ROS production, lipid peroxidation, caspase-3 levels, and DNA fragmentation in PC12 cells stimulated by Aβ, an *in vitro* model of AD (Iuvone et al., 2004; Bedse et al., 2014; Gallelli et al., 2018).

The beneficial effects of CBD were further confirmed by another study where mice were chronically treated (for 3 weeks) with CBD after been injected intracerebroventricularly with fibrillar Aβ (Martín-Moreno et al., 2011). CBD counteracts the Aβ-induced microglial activation, the production of proinflammatory cytokine tumor necrosis factor α (TNF-α) and ameliorates the memory alterations observed in a spatial memory task (Martín-Moreno et al., 2011).

Moreover, Aβ can gradually accumulate in mitochondria, where it can cause reduction of both activity of the respiratory chain complexes and the rate of oxygen consumption leading to a free radical generation and oxidative damage (Caspersen et al., 2005; Lin and Beal, 2006; Manczak et al., 2006; Cassano et al., 2012). To this regard, CBD is able to counteract mitochondrial alterations by the reduction of ROS production induced by both the Aβ and nicotinamide adenine dinucleotide phosphate, reduced form (NADPH) oxidase (NOX) (Hao et al., 2015; Vallée et al., 2017).

It is well know that tau hyperphosphorylation, mostly at serine (Ser) or threonine (Thr) residues, plays a crucial role in the pathogenesis of AD, thereby molecules that reduce phospho-tau aggregates may represent a good candidate for the AD treatment. To this regard, it has been demonstrated that CBD reduces the expression of genes, which encode kinases (GSK-3β, CMK, and MAPK) responsible for aberrant tau phosphorylation, leading to a reduction of tau hyperphosphorylation and subsequent NFT formation (Libro et al., 2016). Likewise, CBD activates the PI3K/Akt signaling through the TRPV1, which is able to inhibit the kinase GSK-3β, thereby decreasing tau phosphorylation (Libro et al., 2016). Finally, CBD downregulates β- and γ-secretase genes leading to a reduction of Aβ production (Libro et al., 2016).

## Conclusion

The present review provided evidence that the nonpsychoactive phytocannabinoids CBD could be a potential pharmacological tool for the treatment of neurodegenerative disorders; its excellent safety and tolerability profile in clinical studies renders it a promising therapeutic agent.

The molecular mechanisms associated with CBD's improvement in PD and AD are likely multifaceted, and although CBD may act on different molecular targets all the beneficial effects are in some extent linked to its antioxidant and anti-inflammatory profile, as observed in *in vitro* and *in vivo* studies. Therefore, this review describes evidence to prove the therapeutical efficacy of CBD in patients affected by neurodegenerative disorders and promotes further research in order to better elucidate the molecular pathways involved in the therapeutic potential of CBD.

## Author Contributions

All authors have contributed to the writing, design, and preparation of figures. The senior authors TC and GS have carried out coordination of efforts.

## Funding

This article was published with a contribution from the University of Foggia.

## Conflict of Interest

The authors declare that the research was conducted in the absence of any commercial or financial relationships that could be construed as a potential conflict of interest.

The reviewer GB declared a past co-authorship with one of the authors TC to the handling editor.
